# Blue light calling: ZmFKF1a and ZmGI1 team up to trigger maize flowering

**DOI:** 10.1093/plcell/koaf203

**Published:** 2025-08-16

**Authors:** Regina Mencia

**Affiliations:** Assistant Features Editor, The Plant Cell, American Society of Plant Biologists; Instituto de Agrobiotecnología del Litoral (CONICET-UNL), Cátedra de Biología Celular y Molecular, Facultad de Bioquímica y Ciencias Biológicas, Universidad Nacional del Litoral, Santa Fe 3000, Argentina

Maize has been part of the human diet for the past 9,000 years. Originally from the tropics, maize has been extensively cultivated and selectively bred, leading to adaptations that now allow it to grow in higher-latitude regions. Despite its rich genetic diversity, tropical maize still struggles to flower in temperate zones, leading to excessive vegetative growth and yield loss (Teixeira et al. 2015). Understanding exactly how maize regulates photoperiod sensing and flowering is of great interest, not only to decipher natural mechanisms but also to exploit them for crop improvement, enabling cultivation across diverse latitudes.

Light is a key environmental factor regulating flowering in plants. Plants can perceive the quality of light and translate it into different responses, including flowering, growth, and other developmental processes (Roeder et al. 2025). In new work, **Wu, Kang, Liu, and collaborators** (Wu et al. 2025) used overexpression and knockout mutants to establish that the FLAVIN-BINDING KELCH REPEAT F-BOX1 (ZmFKF1a) protein acts as a positive regulator of floral transition and is a key component in photoperiod sensing in maize.

Expression of *ZmFKF1a* is tightly linked to day length, always peaking before dusk, and is dependent on blue light. Using yeast 1-hybrid screening, the authors identified the transcription factor ZmLUX2 as a key regulator of *ZmFKF1a* expression, controlling its characteristic diurnal expression pattern. Thus, ZmLUX2 not only regulates flowering through its known role in repressing floral suppressor genes (Zhao et al. 2023) but also by upregulating *ZmFKF1a*. But how does ZmFKF1a accomplish its role in photoperiod flowering regulation? Immunoprecipitation mass spectrometry demonstrated that ZmFKF1a interacts with the clock component GIGANTEA (ZmGI1), an interaction favored by blue light. By interacting with ZmGI1, ZmFKF1a acts to protect ZmGI1 from degradation.

For ZmGI1 to function properly, its partitioning between the nucleus and cytoplasm is essential. The authors demonstrated that blue light illumination promotes the accumulation of the ZmFKF1a–ZmGI1 complex in the nucleus, allowing the activation of floral regulators such as *ZMM4* and ultimately inducing flowering. Interestingly, ZmGI1 has been previously reported as a floral repressor under long-day conditions (Bendix et al. 2013). However, in this study, the authors observed that overexpression of *ZmGI1* in photoperiod-neutral maize lines under long days actually promoted early flowering. This suggests a more complex role for ZmGI1, possibly influenced by genetic background or environmental conditions. Furthermore, in experiments involving overexpression and knockout of either *ZmFKF1a* or *ZmGI1*, mutual regulation was observed: the absence of one protein led to decreased accumulation of its interactor. This indicates that both proteins help maintain each other's steady-state levels.

Now, could the role of ZmFKF1a in photoperiod sensing be exploited for breeding? The authors generated transgenic maize lines overexpressing ZmFKF1a in photoperiod-sensitive tropical inbred lines (CML202) and photoperiod-neutral temperate lines (Huangzao4, HZ4). All plants exhibited early flowering and, surprisingly, the CML202 inbred line also showed an increase in grain weight per ear, highlighting its potential for crop improvement.

Taken together, these findings support a working model for flowering regulation in photoperiod-neutral temperate maize lines (Fig). In this model, blue light promotes the interaction between ZmFKF1a and ZmGI1, facilitating the recruitment of ZmGI1 into the nucleus while protecting it from degradation. The nuclear accumulation of ZmGI1 enhances the transcriptional activation of *ZMM4*, thereby accelerating shoot apex development and flowering. Additionally, the rhythmic expression of *ZmFKF1a* is precisely controlled by the Evening Complex component ZmLUX2 in response to day length, further fine-tuning the photoperiodic flowering response.

**Figure.**
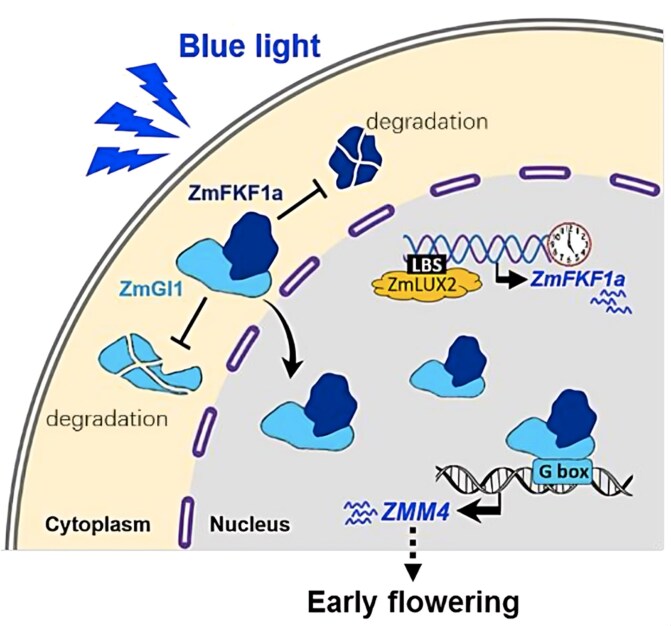
A proposed model shows how ZmFKF1a-ZmGI1 promotes early flowering in maize. ZmLUX2 controls ZmFKF1a expression, enhanced by blue light, which also stabilizes both proteins and enables ZmGI1 nuclear accumulation, activating *ZMM4* to accelerate flowering. Adapted from Wu et al. 2025, Figure 6.

## Recent related articles in *The Plant Cell*

- Dupuis et al. (2025) reported that Chlamydomonas maintains robust diurnal gene expression regardless of light intensity yet adapts photosynthetic efficiency, pigment profiles, and photoprotective mechanisms, suggesting a cellular memory of daytime light conditions and anticipating future environmental light changes.
- Liu et al. (2024) studied how floral evocation and anthesis are distinct events coordinated by a CiPRR7–CiAGL24 module in chrysanthemum flowering in short-day photoperiod.
- Romero et al. (2024) reviewed how plants sense seasonal changes through photoperiodism, focusing on CONSTANS (CO) as a central regulator linking daylength to flowering, senescence, seed size, and circadian rhythms, highlighting its structure, function, and evolutionary adaptations.
- Romero-Lozada et al. (2025) revealed photoperiod-dependent rhythms in transcripts, proteins, and physiological processes, uncovering dynamic plastic orchestration and temporal offsets critical for environmental adaptation in *Ostreococcus tauri*.

## Data Availability

No data were generated in this study.
